# Identificação de Fenótipos de Hipertensão entre os Sexos: Um Estudo de Vida Real com 7.852 Pacientes em Tratamento

**DOI:** 10.36660/abc.20250037

**Published:** 2025-08-22

**Authors:** Eduardo Costa Duarte Barbosa, Sérgio Kakuta Kato, Audes Feitosa, Marco Antonio Mota-Gomes, Roberto Dischinger Miranda, Andréa Araujo Brandão, Weimar Kunz Sebba Barroso, Bruna Eibel

**Affiliations:** 1 Serviço de Hipertensão e Cariometabolismo Hospital São Francisco Santa Casa de Miseticórdia de Porto Alegre Porto Alegre RS Brasil Serviço de Hipertensão e Cariometabolismo do Hospital São Francisco da Santa Casa de Miseticórdia de Porto Alegre, Porto Alegre, RS – Brasil; 2 Universidade Feevale Novo Hamburgo RS Brasil Universidade Feevale, Novo Hamburgo, RS – Brasil; 3 Universidade Federal de Ciências da Saúde de Porto Alegre Porto Alegre RS Brasil Universidade Federal de Ciências da Saúde de Porto Alegre, Porto Alegre, RS – Brasil; 4 Pronto Socorro Cardiológico Universitário de Pernambuco Recife PE Brasil Pronto Socorro Cardiológico Universitário de Pernambuco – PROCAPE, Recife, PE – Brasil; 5 Centro Universitário CESMAC Hospital do Coração Maceió AL Brasil Centro Universitário CESMAC - Hospital do Coração, Maceió, AL – Brasil; 6 Disciplina de Geriatria e Gerontologia Escola Paulista de Medicina Universidade Federal de São Paulo São Paulo SP Brasil Setor de Doenças Cardiovasculares, Disciplina de Geriatria e Gerontologia, Escola Paulista de Medicina, Universidade Federal de São Paulo (UNIFESP), São Paulo, SP – Brasil; 7 Universidade do Estado do Rio de Janeiro Rio de Janeiro RJ Brasil Universidade do Estado do Rio de Janeiro, Rio de Janeiro, RJ – Brasil; 8 Faculdade de Medicina Unidade de Hipertensão Arterial Universidade Federal de Goiás Goiânia GO Brasil Programa de Pós-graduação Ciências da Saúde, Faculdade de Medicina, Unidade de Hipertensão Arterial, Universidade Federal de Goiás, Goiânia, GO – Brasil; 9 Instituto de Cardiologia Fundação Universitária de Cardiologia Porto Alegre RS Brasil Instituto de Cardiologia/Fundação Universitária de Cardiologia, Porto Alegre, RS – Brasil

**Keywords:** Hipertensão, Pressão Arterial, Fenótipo

## Abstract

**Fundamento:**

Embora os limiares de pressão arterial (PA) estejam bem definidos, há escassez de dados sobre os possíveis fenótipos de hipertensão arterial entre os sexos.

**Objetivos:**

Identificar os fenótipos de hipertensão arterial em homens e mulheres com hipertensão em tratamento.

**Métodos:**

Foram incluídos adultos com hipertensão arterial, entre 18 e 80 anos, em tratamento medicamentoso, recrutados oportunisticamente em diversas regiões do Brasil. As avaliações incluíram medidas de PA em consultório e por monitorização residencial da PA (MRPA). Foram considerados quatro fenótipos de hipertensão arterial: i) hipertensão controlada: PA < 140/90 mmHg e MRPA < 130/80 mmHg; ii) hipertensão do avental branco não controlada: PA ≥ 140/90 mmHg e MRPA < 130/80 mmHg; iii) hipertensão mascarada não controlada: PA < 140/90 mmHg e MRPA ≥ 130/80 mmHg; iv) hipertensão sustentada não controlada: PA ≥ 140/90 mmHg e MRPA ≥ 130/80 mmHg. O nível de significância adotado foi de 5% (p < 0,05).

**Resultados:**

Dos 7.852 pacientes avaliados, todos em uso de medicação anti-hipertensiva, 3.162 (40,3%) apresentaram hipertensão controlada, sendo 1.115 (37,6%) homens e 2.047 (41,9%) mulheres (p < 0,001); 675 (8,6%) apresentaram hipertensão do avental branco não controlada, sendo 217 (7,3%) homens e 458 (9,4%) mulheres (p < 0,001); 1.605 (20,4%) apresentaram hipertensão mascarada não controlada, sendo 645 (21,7%) homens e 960 (19,7%) mulheres (p < 0,001); e 2.410 (30,7%) apresentaram hipertensão sustentada não controlada, sendo 992 (33,4%) homens e 1.418 (29%) mulheres (p < 0,001).

**Conclusões:**

Este é o primeiro estudo populacional brasileiro a avaliar fenótipos de hipertensão por sexo. As mulheres apresentaram melhor controle pressórico em comparação aos homens, tanto em consultório quanto em ambiente domiciliar.

## Graphical abstract

**Figura Central:**
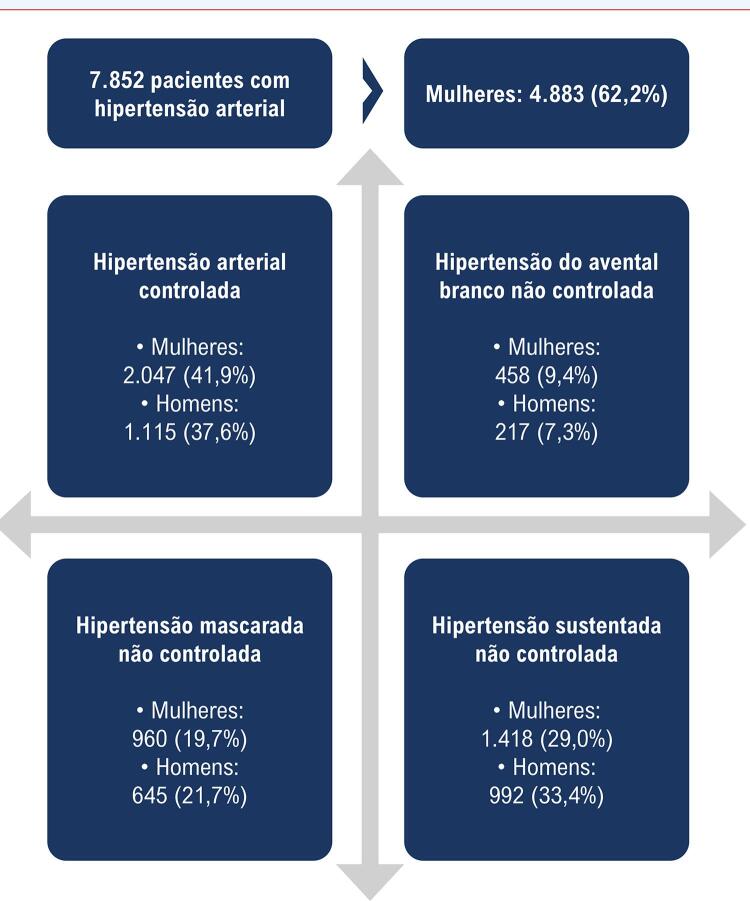
XIdentificação de Fenótipos de Hipertensão entre os Sexos: Um Estudo de Vida Real com 7.852 Pacientes em Tratamento

## Introdução

A pressão arterial (PA) elevada é um dos principais fatores de risco para a carga global de doenças.^1^ As doenças cardiovasculares (DCVs), especialmente o acidente vascular cerebral e a doença arterial coronariana, têm sido a principal causa de mortalidade no Brasil nas últimas décadas. Entre as DCVs, a doença arterial coronariana é a mais letal, respondendo por 31% dos óbitos por causas cardiovasculares, seguida pelas doenças cerebrovasculares (30%), pela cardiopatia hipertensiva (14%) e por outras formas de doença cardíaca, principalmente a insuficiência cardíaca congestiva (18%).^2^

Existem quatro fenótipos distintos de hipertensão arterial. Os termos hipertensão do avental branco e hipertensão mascarada foram originalmente definidos para indivíduos não tratados; no entanto, esses padrões de variação da PA, dentro e fora do consultório, também ocorrem em pacientes em uso de medicamentos anti-hipertensivos. Para esses casos, adotam-se as seguintes classificações: i) hipertensão controlada (HC), quando a PA está normal tanto no consultório quanto fora dele; ii) hipertensão sustentada não controlada (HSNC), quando a PA está elevada em ambos os ambientes; iii) HAB não controlada (HABNC), quando a PA é elevada no consultório e normal fora dele; e iv) HM não controlada (HMNC), quando a PA é normal no consultório, mas elevada fora dele. As prevalências estimadas no Brasil para esses fenótipos variam entre 31-41% (HC), 28-41% (HSNC), 19-20% (HMNC) e 7-9% (HABNC).^3^

Embora a prevalência varie entre os estudos, a HAB pode ser identificada em aproximadamente 15% a 19% dos indivíduos avaliados em consultório, sendo mais comum em pacientes com hipertensão arterial em estágio 1. Diversos fatores podem contribuir para elevações da PA fora do consultório em comparação às medições no ambiente clínico, dependendo da população analisada. Entre esses fatores estão: idade avançada, sexo masculino, tabagismo, consumo de álcool, prática de atividade física, ansiedade, estresse, obesidade, diabetes melito, doença renal crônica e histórico familiar de hipertensão arterial.^3^

A última diretriz brasileira manteve os critérios anteriores, considerando como hipertenso o indivíduo com PA sistólica (PAS) ≥ 140 mmHg e/ou PA diastólica (PAD) ≥ 90 mmHg em medidas de consultório.^3^ Recomenda-se que a PA no consultório seja categorizada como não elevada, elevada ou hipertensão, a fim de orientar as decisões terapêuticas, conforme as novas diretrizes de 2024 da European Society of Cardiology (ESC) para o tratamento da PA elevada e da hipertensão arterial. O diagnóstico de hipertensão arterial ou PA elevada deve ser confirmado por medições fora do consultório, por meio da monitorização residencial da PA (MRPA) ou da monitorização ambulatorial da PA (MAPA), ou, no mínimo, por uma nova aferição em consultório. Além disso, quando a PA no consultório estiver entre 120-139/70-89 mmHg, o paciente deve ser classificado como portador de PA elevada, sendo recomendada a estratificação do risco de DCV para guiar a conduta. Para pacientes com hipertensão arterial (PA ≥ 140/90 mmHg), considera-se que todos apresentam risco cardiovascular suficientemente alto para se beneficiarem do tratamento farmacológico.^4^

Embora os limiares de PA estejam bem estabelecidos, ainda há uma lacuna de informações sobre os diferentes fenótipos de hipertensão arterial entre os sexos — questão que este estudo se propôs a investigar.^5-8^

## Métodos

### Desenho do estudo e população

Trata-se de um estudo transversal multicêntrico de vida real, baseado no Registro Nacional do Controle da Hipertensão Arterial Avaliado pela Medida de Consultório e Residencial no Brasil (Registro LHAR). Foram incluídos pacientes com hipertensão arterial, homens e mulheres adultos, com idades entre 18 e 80 anos, recrutados oportunisticamente entre 2019 e 2023. A triagem foi realizada em clínicas participantes, localizadas na maioria das regiões do Brasil.

### Aferição da PA

Todos os profissionais responsáveis pela aferição da PA foram previamente treinados e seguiram um protocolo padronizado para garantir a uniformidade das medições. Os participantes realizaram as medições em posição sentada, com leituras feitas em intervalos de 1 minuto.

Tanto as medidas de PA em consultório quanto as da MRPA foram obtidas com o equipamento eletrônico HEM-7320 (OMRON HEALTHCARE Co., Ltd. 53, Kyoto, Japão). O protocolo da MRPA incluiu duas medições no consultório e seis medições domiciliares por dia (três pela manhã e três à tarde/noite), durante 4 dias consecutivos.^9^ A primeira aferição (dia 1) foi realizada no consultório, com o objetivo de demonstrar o procedimento ao paciente ou acompanhante. As aferições domiciliares ocorreram nos dias 2, 3, 4 e 5.

As leituras da MRPA foram enviadas para a plataforma TeleMRPA (www.telemrpa.com) para análise e emissão dos laudos. Para as análises, foram consideradas as médias da PAS e da PAD.

### Variáveis coletadas

Também foi aplicado um questionário para coleta de dados demográficos, presença de comorbidades, fatores de risco relacionados ao estilo de vida e uso de medicamentos anti-hipertensivos. O índice de massa corporal (IMC) dos participantes variou entre 18,5 kg/m^2^ (peso normal) e 40 kg/m^2^ (obesidade grau III).

Foram considerados os seguintes fenótipos de hipertensão arterial: i) HC: PA < 140/90 mmHg e MRPA < 130/80 mmHg; ii) HABNC: PA ≥ 140/90 mmHg e MRPA < 130/80 mmHg; iii) HMNC: PA < 140/90 mmHg e MRPA ≥ 130/80 mmHg; iv) HSNC: PA ≥ 140/90 mmHg e MRPA ≥ 130/80 mmHg.

### Aspectos éticos

Os dados foram coletados localmente por meio de formulários em papel e posteriormente enviados à equipe central para tabulação e análise. Todos os participantes leram e assinaram o Termo de Consentimento Livre e Esclarecido. O estudo foi aprovado pelo Comitê de Ética em Pesquisa da Universidade Federal de Goiás (CAAE: 08208619.8.0000.5078), em conformidade com a Resolução nº 466/2012 do Conselho Nacional de Saúde.

### Análise estatística

As variáveis qualitativas foram descritas por frequências absolutas e relativas, enquanto as variáveis quantitativas foram apresentadas como média e desvio padrão, uma vez que não houve violação da suposição de normalidade, avaliada pelo teste de Kolmogorov-Smirnov. A associação geral entre os fenótipos de hipertensão arterial e o sexo foi analisada pelo teste do qui-quadrado, com complementação por análise residual ajustada, a fim de identificar associações específicas entre cada fenótipo e o sexo. Para cada sexo, a comparação entre a PA média de consultório e a PA média domiciliar foi realizada por meio do teste *t* de Student para amostras pareadas. As análises estatísticas foram realizadas no IBM SPSS Statistics for Windows, versão 24 (IBM Corp., Armonk, N.Y., USA). O nível de significância de 5% (p < 0,05) foi adotado.

## Resultados

No total, foram avaliados 7.852 pacientes em uso de medicação anti-hipertensiva em ambiente ambulatorial, dos quais 4.883 (62,2%) eram do sexo feminino. A média de idade e o IMC das mulheres foram de 61,4 ± 12,6 anos e 28,2 ± 4,4 kg/m^2^, respectivamente, enquanto nos homens foram de 58,7 ± 12,8 anos e 28,7 ± 4,0 kg/m^2^, com diferença estatisticamente significativa entre os sexos para ambas as variáveis (p < 0,001).

Quanto aos fenótipos de hipertensão arterial, observou-se que 3.162 (40,3%) apresentaram HC, 675 (8,6%) HABNC, 1.605 (20,4%) HMNC e 2.410 (30,7%) HSNC. A distribuição dos fenótipos por sexo e o comportamento da hipertensão arterial estão detalhados na Tabela 1 e na Figura Central.

**Tabela 1:** – Distribuição dos fenótipos de hipertensão arterial por sexo

Fenótipos de hipertensão arterial	Homens, n (%)	Mulheres, n (%)	Valor p
HC	1.115 (37,6%)	2.047 (41,9%)	<0,001*
HABNC	217 (7,3%)	458 (9,4%)	<0,001*
HMNC	645 (21,7%)	960 (19,7%)	<0,001*
HSNC	992 (33,4%)	1.418 (29,0%)	<0,001*

Frequências absolutas e relativas. Teste qui-quadrado; *nível de significância: p<0,05. HC: hipertensão controlada; HABNC: hipertensão do avental branco não controlada; HMNC: hipertensão mascarada não controlada; HSNC: hipertensão sustentada não controlada.

Além disso, foi realizada a comparação entre as médias de PAS e PAD de consultório e as médias de PAS e PAD domiciliares, estratificada por sexo, conforme apresentado na Tabela 2.

**Tabela 2:** – Comparação entre PA de consultório e domiciliar por sexo

Sexo	Local de verificação	PAS (mmHg)	PAD (mmHg)	Valor p
Homens (n = 2.969)	Consultório	132 ± 17	83 ± 11	<0,001*
	Domicílio	126 ± 13	78 ± 9	<0,001*
Mulheres (n = 4.883)	Consultório	129 ± 19	82 ± 11	<0,001*
	Domicílio	122 ± 15	77 ± 9	<0,001*

Valores expressos em média ± desvio padrão. Teste t para amostras pareadas; *nível de significância: p<0,05. PA: pressão arterial; PAD: PA diastólica; PAS: PA sistólica.

A Tabela 3 apresenta a distribuição dos fenótipos de hipertensão arterial entre homens e mulheres nas diferentes regiões do Brasil, evidenciando a representatividade nacional da amostra. As regiões Nordeste, Sudeste e Sul apresentaram, respectivamente, as maiores frequências de HC em ambos os sexos. A região Nordeste também apresentou a maior frequência de HABNC, tanto em homens quanto em mulheres. Além disso, as regiões Nordeste, Sudeste e Sul registraram, nessa ordem, as maiores prevalências de HMNC e HSNC, igualmente em ambos os sexos.

**Tabela 3:** – Distribuição dos fenótipos de hipertensão arterial por sexo e região brasileira

Região	HC		HABNC		HMNC		HSNC	
	Homens, n (%)	Mulheres, n (%)	Homens, n (%)	Mulheres, n (%)	Homens, n (%)	Mulheres, n (%)	Homens, n (%)	Mulheres, n (%)
Centro-Oeste	37 (3,3%)	54 (2,6%)	4 (1,8%)	16 (3,5%)	18 (2,8%)	31 (3,2%)	31 (3,1%)	36 (2,5%)
Nordeste	525 (47,1%)	947 (46,3%)	101 (46,5%)	222 (48,5%)	246 (38,1%)	342 (35,6%)	376 (37,9%)	560 (39,5%)
Norte	14 (1,3%)	20 (1,0%)	6 (2,8%)	12 (2,6%)	7 (1,1%)	7 (0,7%)	9 (0,9%)	27 (1,9%)
Sudeste	303 (27,2%)	555 (27,1%)	60 (27,6%)	126 (27,5%)	225 (34,9%)	317 (33,0%)	340 (34,3%)	478 (33,7%)
Sul	233 (20,9%)	465 (22,7%)	46 (21,2%)	79 (17,2%)	147 (22,8%)	258 (26,9%)	230 (23,2%)	311 (21,9%)

Frequências absolutas e relativas. HC: hipertensão controlada; HABNC: hipertensão do avental branco não controlada; HMNC: hipertensão mascarada não controlada; HSNC: hipertensão sustentada não controlada.

## Discussão

Nossos resultados corroboram as recomendações das Diretrizes Brasileiras de Medidas da Pressão Arterial Dentro e Fora do Consultório – 2023,^5^ ao reforçar a importância da aferição da PA tanto em consultório quanto em ambiente domiciliar. Na amostra analisada, observou-se uma proporção relativamente pequena de pacientes com HABNC, predominantemente do sexo feminino, em concordância com os achados de Mancia et al. (2022).^6^ Destaca-se, contudo, o elevado percentual de indivíduos com HSNC, um achado semelhante ao relatado por Spatz et al. (2019).^7^ As mulheres apresentavam maior média de idade, porém menor IMC em comparação aos homens. No entanto, ambos os sexos estavam, majoritariamente, na faixa etária de meia-idade e apresentavam sobrepeso — fatores de risco bem estabelecidos para o desenvolvimento de hipertensão arterial.^8^

Em concordância com nossos achados, o Estudo Longitudinal da Saúde dos Idosos Brasileiros (ELSI-Brasil) identificou que 59,9% da amostra era composta por mulheres, com faixa etária semelhante à observada neste estudo. De modo geral, os comportamentos relacionados à saúde contribuíram mais significativamente para o controle da hipertensão arterial entre as mulheres (66,3%) do que entre os homens (36,2%).^9^

Ao comparar a PA de consultório com a PA domiciliar em cada sexo, observou-se que a maioria dos participantes apresentava HC. No entanto, nosso estudo também evidenciou a presença de três fenótipos menos frequentemente relatados: HABNC, HMNC e HSNC. Esses padrões merecem atenção clínica e validação conceitual, reforçando a importância da MRPA como ferramenta essencial para um diagnóstico mais preciso e para a definição de um tratamento personalizado.^10^

A HMNC apresentou prevalência de aproximadamente 20% em nossa amostra. Assim como ocorre com a HABNC, a prevalência da HMNC pode variar entre diferentes populações. No entanto, estima-se que em geral ela seja detectada em 7-9% dos indivíduos avaliados em consultório, podendo alcançar até 15% em determinados grupos.^3^

Nosso estudo incluiu uma ampla amostra de pacientes avaliados em consultório, seguindo as diretrizes vigentes para o monitoramento da PA e realizando a comparação com as medidas domiciliares.^11^ No entanto, não foi possível assegurar que todas as medições domiciliares tenham seguido rigorosamente as recomendações estabelecidas para aferição da PA em consultório, apesar das orientações fornecidas para a realização da MRPA. Trata-se do primeiro estudo populacional brasileiro a avaliar os fenótipos de hipertensão arterial segundo o sexo. Contudo, não foram investigadas questões relacionadas ao gênero, como o uso de hormônios e suas possíveis implicações nos resultados. Por fim, destaca-se a representatividade da população estudada, bem como sua ampla distribuição geográfica.

## Conclusões

Este é o primeiro estudo populacional brasileiro a avaliar os fenótipos de hipertensão arterial segundo o sexo. As mulheres apresentaram menores níveis de PA, tanto em consultório quanto em ambiente domiciliar, em comparação aos homens. Além disso, demonstraram melhor controle da hipertensão arterial, com maior prevalência dos fenótipos HC e HABNC, enquanto os homens apresentaram, com maior frequência, os fenótipos HSNC e HMNC.
