# Supplementary material for: Identificação de Fenótipos de Hipertensão entre os Sexos: Um Estudo de Vida Real com 7.852 Pacientes em Tratamento
Source: Arq Bras Cardiol. 2025 Aug 22;122(9):e20250037. [Article in Portuguese] doi: 10.36660/abc.20250037 (PMC12677911; doi:10.36660/abc.20250037)
Supplement: Translation

# Translation: Identification of Hypertension Phenotypes by Sex: A Real-World Study of 7,852 Treated Patients

**DOI:** 10.36660/abc.20250037i

**Authors:** Eduardo Costa Duarte Barbosa, Sérgio Kakuta Kato, Audes Feitosa, Marco Antonio Mota-Gomes, Roberto Dischinger Miranda, Andréa Araujo Brandão, Weimar Kunz Sebba Barroso, Bruna Eibel

**Affiliations:** 1 Brasil Serviço de Hipertensão e Cariometabolismo do Hospital São Francisco da Santa Casa de Miseticórdia de Porto Alegre, Porto Alegre, RS – Brasil; 2 Brazil Universidade Feevale, Novo Hamburgo, RS – Brazil; 3 Brazil Universidade Federal de Ciências da Saúde de Porto Alegre, Porto Alegre, RS – Brazil; 4 Brazil Pronto Socorro Cardiológico Universitário de Pernambuco – PROCAPE, Recife, PE – Brazil; 5 Brazil Centro Universitário CESMAC - Hospital do Coração, Maceió, AL – Brazil; 6 Brazil Setor de Doenças Cardiovasculares, Disciplina de Geriatria e Gerontologia, Escola Paulista de Medicina, Universidade Federal de São Paulo (UNIFESP), São Paulo, SP – Brazil; 7 Brazil Universidade do Estado do Rio de Janeiro, Rio de Janeiro, RJ – Brazil; 8 Brazil Programa de Pós-graduação Ciências da Saúde, Faculdade de Medicina, Unidade de Hipertensão Arterial, Universidade Federal de Goiás, Goiânia, GO – Brazil; 9 Brazil Instituto de Cardiologia/Fundação Universitária de Cardiologia, Porto Alegre, RS – Brazil

**Keywords:** Hypertension, Arterial Pressure, Phenotype

## Abstract

**Background:**

Although blood pressure (BP) thresholds are well established, there is a lack of data on potential hypertension phenotypes across sexes.

**Objectives:**

To identify hypertension phenotypes in men and women undergoing antihypertensive treatment.

**Methods:**

Adults aged 18 to 80 years with diagnosed hypertension and undergoing pharmacological treatment were opportunistically recruited from various regions of Brazil. Assessments included office BP measurements and home blood pressure monitoring (HBPM). Four hypertension phenotypes were defined: (i) controlled hypertension: office BP < 140/90 mmHg and HBPM < 130/80 mmHg; (ii) white-coat uncontrolled hypertension: office BP ≥ 140/90 mmHg and HBPM < 130/80 mmHg; (iii) masked uncontrolled hypertension: office BP < 140/90 mmHg and HBPM ≥ 130/80 mmHg; (iv) sustained uncontrolled hypertension: office BP ≥ 140/90 mmHg and HBPM ≥ 130/80 mmHg. A significance level of 5% (p < 0.05) was adopted.

**Results:**

Among the 7,852 patients on antihypertensive therapy, 3,162 (40.3%) had controlled hypertension, including 1,115 (37.6%) men and 2,047 (41.9%) women (p < 0.001); 675 (8.6%) had white-coat uncontrolled hypertension, with 217 (7.3%) men and 458 (9.4%) women (p < 0.001); 1,605 (20.4%) had masked uncontrolled hypertension, including 645 (21.7%) men and 960 (19.7%) women (p < 0.001); and 2,410 (30.7%) had sustained uncontrolled hypertension, including 992 (33.4%) men and 1,418 (29%) women (p < 0.001).

**Conclusions:**

This is the first Brazilian population-based study to assess hypertension phenotypes by sex. Women demonstrated better BP control than men, both in clinical settings and at home.

## Introduction

Elevated blood pressure (BP) is one of the leading risk factors for the global burden of disease.^1^ Cardiovascular diseases (CVDs), particularly stroke and coronary artery disease, have been the primary cause of mortality in Brazil over recent decades. Among CVDs, coronary artery disease is the most lethal, accounting for 31% of cardiovascular deaths, followed by cerebrovascular diseases (30%), hypertensive heart disease (14%), and other forms of heart disease, mainly congestive heart failure (18%).^2^

There are four distinct phenotypes of arterial hypertension. The terms white-coat hypertension and masked hypertension were originally defined in untreated individuals. However, these patterns of BP variation, both inside and outside the clinical setting, also occur in patients receiving antihypertensive therapy. In such cases, the following classifications are used: (i) controlled hypertension (CH), when BP is normal both in and out of the office; (ii) sustained uncontrolled hypertension (SUH), when BP is elevated in both settings; (iii) white-coat uncontrolled hypertension (WUCH), when BP is elevated in the office but normal outside; and (iv) masked uncontrolled hypertension (MUCH), when BP is normal in the office but elevated outside. In Brazil, the estimated prevalence of these phenotypes ranges from 31-41% (CH), 28-41% (SUH), 19-20% (MUCH), and 7-9% (WUCH).^3^

Although prevalence rates vary across studies, WUCH can be identified in approximately 15% to 19% of individuals evaluated in the clinical setting and is more common among patients with stage 1 hypertension. Several factors may contribute to elevated BP outside the office compared to clinical measurements, depending on the population studied. These factors include older age, male sex, smoking, alcohol consumption, physical activity, anxiety, stress, obesity, diabetes mellitus, chronic kidney disease, and a family history of hypertension.^3^

The most recent Brazilian guideline retained the previous criteria, defining hypertension as a systolic BP (SBP) ≥ 140 mmHg and/or a diastolic BP (DBP) ≥ 90 mmHg based on office measurements.^3^ Office BP be categorized as normal, elevated, or hypertensive to guide therapeutic decisions in accordance with the 2024 guidelines of the European Society of Cardiology (ESC) for the management of elevated BP and arterial hypertension. The diagnosis of hypertension or elevated BP should be confirmed through out-of-office measurements, using home BP monitoring (HBPM) or ambulatory BP monitoring (ABPM), or at a minimum, through repeat office measurements. In addition, when office BP falls within the range of 120-139/70-89 mmHg, the patient should be classified as having elevated BP, and cardiovascular risk stratification is recommended to guide clinical management. For individuals with hypertension (BP ≥ 140/90 mmHg), all are considered to be at sufficiently high cardiovascular risk to benefit from pharmacological treatment.^4^

Although BP thresholds are well established, there remains a gap in knowledge regarding the distribution of hypertension phenotypes across sexes — a question this study aims to address.^5-8^

## Methods

### Study design and population

This was a real-world, multicenter, cross-sectional study based on the National Registry of Hypertension Control Evaluated by Office and Home Measurements (The LHAR National Registry). The study included adult men and women aged 18 to 80 years with a diagnosis of hypertension, opportunistically recruited between 2019 and 2023. Screening was conducted at participating clinics located in most regions of Brazil.

### BP measurement

All professionals responsible for BP measurement were previously trained and followed a standardized protocol to ensure consistency across readings. Participants underwent measurements in the seated position, with readings taken at 1-minute intervals.

Both office BP and HBPM readings were obtained using the electronic device HEM-7320 (OMRON HEALTHCARE Co., Ltd., Kyoto, Japan). The HBPM protocol included two office measurements and six home measurements per day (three in the morning and three in the afternoon/evening) over 4 consecutive days.^9^ The first measurement (Day 1) was performed in the office to demonstrate the procedure to the patient or caregiver. Home measurements were conducted on Days 2, 3, 4, and 5.

HBPM readings were submitted to the TeleMRPA platform (www.telemrpa.com) for analysis and report generation. For analysis purposes, the average systolic (SBP) and diastolic (DBP) BP values were considered.

### Collected variables

A questionnaire was also administered to collect demographic data, the presence of comorbidities, lifestyle-related risk factors, and the use of antihypertensive medications. Participants’ body mass index (BMI) ranged from 18.5 kg/m^2^ (normal weight) to 40 kg/m^2^ (class III obesity).

The following hypertension phenotypes were considered: i) CH: BP < 140/90 mmHg and HBPM < 130/80 mmHg; ii) WUCH: BP ≥ 140/90 mmHg and HBPM < 130/80 mmHg; iii) MUCH: BP < 140/90 mmHg and HBPM ≥ 130/80 mmHg; iv) SUH: BP ≥ 140/90 mmHg and HBPM ≥ 130/80 mmHg.

### Ethical considerations

Data were collected locally using paper forms and subsequently sent to the central research team for tabulation and analysis. All participants read and signed the informed consent form. The study was approved by the Research Ethics Committee of the Universidade Federal de Goiás (CAAE: 08208619.8.0000.5078) in accordance with Resolution No. 466/2012 of the Brazilian National Health Council.

### Statistical analysis

Qualitative variables were described using absolute and relative frequencies, while quantitative variables were presented as mean and standard deviation, as the assumption of normality was not violated, according to the Kolmogorov–Smirnov test. The overall association between hypertension phenotypes and sex was analyzed using the chi-square test, supplemented by adjusted residual analysis to identify specific associations between each phenotype and sex. For each sex, comparisons between mean office BP and mean home BP were performed using the paired Student’s *t*-test. Statistical analyses were conducted using IBM SPSS Statistics for Windows, version 24 (IBM Corp., Armonk, N.Y., USA). A significance level of 5% (p < 0.05) was adopted.

## Results

A total of 7,852 patients receiving antihypertensive medication in an outpatient setting were evaluated, of whom 4,883 (62.2%) were female. The mean age and BMI of women were 61.4 ± 12.6 years and 28.2 ± 4.4 kg/m^2^, respectively, while for men, the values were 58.7 ± 12.8 years and 28.7 ± 4.0 kg/m^2^. There was a statistically significant difference between sexes for both variables (p < 0.001).

Regarding hypertension phenotypes, 3,162 patients (40.3%) presented with CH, 675 (8.6%) with WUCH, 1,605 (20.4%) with MUCH, and 2,410 (30.7%) with SUH. The distribution of phenotypes by sex and the overall pattern of hypertension are detailed in Table 1 and Central Illustration.

**Table 1:** – Distribution of hypertension phenotypes by sex

Hypertension phenotypes	Men, n (%)	Women, n (%)	p-value
CH	1,115 (37.6%)	2,047 (41.9%)	<0.001*
WUCH	217 (7.3%)	458 (9.4%)	<0.001*
MUCH	645 (21.7%)	960 (19.7%)	<0.001*
SUH	992 (33.4%)	1,418 (29.0%)	<0.001*

Absolute and relative frequencies. Chi-square test; *significance level: p < 0.05. CH: controlled hypertension; MUCH: masked uncontrolled hypertension; SUH: sustained uncontrolled hypertension WUCH: white-coat uncontrolled hypertension.

Besides, a comparison was performed between mean office SBP and DBP and mean home SBP and DBP, stratified by sex, as presented in Table 2.

**Table 2:** – Comparison between office and home BP by sex

Sex	Measurement setting	SBP (mmHg)	DBP (mmHg)	p-value
Men (n = 2,969)	Office	132 ± 17	83 ± 11	<0.001*
	Home	126 ± 13	78 ± 9	<0.001*
Women (n = 4,883)	Office	129 ± 19	82 ± 11	<0.001*
	Home	122 ± 15	77 ± 9	<0.001*

Values expressed as mean ± standard deviation. Paired Student’s t-test; *significance level: p < 0.05. BP: blood pressure; DBP: diastolic blood pressure; SBP: systolic blood pressure.

Table 3 presents the distribution of hypertension phenotypes among men and women across different regions of Brazil, highlighting the national representativeness of the sample. The Northeast, Southeast, and South regions showed the highest frequencies of CH in both sexes, respectively. The Northeast region also had the highest frequency of WUCH among both men and women. Additionally, the Northeast, Southeast, and South regions, in that order, recorded the highest prevalences of MUCH and SUH, also in both sexes.

**Table 3:** – Distribution of hypertension phenotypes by sex and Brazilian region

Region	CH		WUCH		MUCH		SUH	
	Men, n (%)	Women, n (%)	Men, n (%)	Women, n (%)	Men, n (%)	Women, n (%)	Men, n (%)	Women, n (%)
Central-West	37 (3.3%)	54 (2.6%)	4 (1.8%)	16 (3.5%)	18 (2.8%)	31 (3.2%)	31 (3.1%)	36 (2.5%)
Northeast	525 (47.1%)	947 (46.3%)	101 (46.5%)	222 (48.5%)	246 (38.1%)	342 (35.6%)	376 (37.9%)	560 (39.5%)
North	14 (1.3%)	20 (1.0%)	6 (2.8%)	12 (2.6%)	7 (1.1%)	7 (0.7%)	9 (0.9%)	27 (1.9%)
Southeast	303 (27.2%)	555 (27.1%)	60 (27.6%)	126 (27.5%)	225 (34.9%)	317 (33.0%)	340 (34.3%)	478 (33.7%)
South	233 (20.9%)	465 (22.7%)	46 (21.2%)	79 (17.2%)	147 (22.8%)	258 (26.9%)	230 (23.2%)	311 (21.9%)

Absolute and relative frequencies. CH: controlled hypertension; MUCH: masked uncontrolled hypertension; SUH: sustained uncontrolled hypertension; WUCH: white-coat uncontrolled hypertension.

## Discussion

Our findings are consistent with the recommendations of the Brazilian Guidelines for In-office and Out-of-office Blood Pressure Measurement – 2023,^5^ which reinforces the importance of assessing BP both in the office and at home. In our sample, a relatively small proportion of patients had WUCH, predominantly women, which aligns with the findings of Mancia et al. (2022).^6^ Notably, a high percentage of individuals presented with SUH, a finding similar to that reported by Spatz et al. (2019).^7^ The mean age of women was higher than that of men, but their BMI was lower. Nonetheless, both sexes were predominantly middle-aged and classified as overweight — well-established risk factors for the development of hypertension.^8^

In line with our findings, the Brazilian Longitudinal Study of Aging (ELSI-Brazil) reported that 59.9% of the sample consisted of women, with an age range similar to that observed in the present study. Overall, health-related behaviors contributed more significantly to hypertension control among women (66.3%) than among men (36.2%).^9^

When comparing office BP with home BP in each sex, most participants were classified as having CH. However, our study also revealed the presence of three less frequently reported phenotypes: WUCH, MUCH, and SUH. These patterns warrant clinical attention and conceptual validation, underscoring the importance of HBPM as an essential tool for more accurate diagnosis and the development of personalized treatment strategies.^10^

MUCH had a prevalence of approximately 20% in our sample. As with WUCH, the prevalence of MUCH may vary across different populations. However, it is generally estimated to be present in 7-9% of individuals assessed in the office, potentially reaching up to 15% in certain groups.^3^

Our study included a large sample of patients evaluated in the office, in accordance with current guidelines for BP monitoring, and compared these measurements with home BP values.^11^ However, it was not possible to ensure that all home measurements strictly followed the standardized recommendations for office BP measurement, despite the guidance provided for HBPM. This is the first Brazilian population-based study to assess hypertension phenotypes by sex. Nonetheless, issues related to gender (e.g., hormone use and its potential implications) were not investigated. Finally, the representativeness of the studied population and its broad geographic distribution should be highlighted.

## Conclusions

This is the first Brazilian population-based study to assess hypertension phenotypes by sex. Women exhibited lower BP levels both in the office and at home compared to men. They also demonstrated better hypertension control, with a higher prevalence of CH and WUCH phenotypes, while men more frequently presented with SUH and MUCH phenotypes.
